# Spontaneous dislocation of a crystalline lens to the anterior chamber with pupillary block glaucoma in Noonan Syndrome: a case report

**DOI:** 10.11604/pamj.2014.17.135.3049

**Published:** 2014-02-26

**Authors:** Udayaditya Mukhopadhyaya, Chandana Chakraborti, Anindita Mondal, Ujjal Pattyanayak, Rajesh Kumar Agarwal, Partha Tripathi

**Affiliations:** 1 Regional Institute Of Ophthalmology(RIO), Kolkata, Kolkata 700073, West Bengal, India; 2 Dept of Ophthalmology, Calcutta National Medical College and Hospital, Kolkata, India; 3 KPC Medical College, Kolkata, India; 4 Chinsurah District Hospital, Hooghly, West Bengal, India

**Keywords:** Noonan Syndrome, dislocated lens, angle closure glaucoma

## Abstract

We report a 13-year-old child with Noonan Syndrome who developed spontaneous dislocation of the crystalline lens in anterior chamber leading to pupillary block glaucoma in the left eye and subluxation of lens in right eye. Intracapsular extraction of the dislocated lens was done in the left eye. Prompt diagnosis and management is needed in such cases to avoid glaucoma and corneal endothelial cell damage. We could not find any such case after thorough Medline search.

## Introduction

Noonan Syndrome (NS) is an autosomal dominant disorder first reported by Koblinski in 1883 and described by Noonan and Ehmke in 1963. The incidence of NS is estimated to be between 1 in 1000 to 1 in 2500 live births. Patients with NS commonly present with variable ophthalmic manifestations such as hypertelorism, ptosis, epicanthal folds, antimongoloid slant, nasolacrimal duct obstruction, refractive error, strabismus, amblyopia, nystagmus, anterior segment changes, and abnormal fundoscopy reported by Lee NB and Reynolds DJ et al [1, 2]. Systemic features are short stature, heart disease, webbed neck, a pectus deformity and cryptorchidism in males. The diagnosis rests solely on clinical findings with normal Karyotyping but a mutation in the PTPN11 gene was found to be present in about 50 -60% of individuals with NS reported by Zenkar M et al [3].

Lens dislocation (ectopia lentis) in children may occur after trauma or in association with ocular or systemic disease reported by Choi DY [4]. Spontaneous subluxation or luxation of a normal lens in a case of NS has not been reported.

## Patient and observation

**Figure 1:**
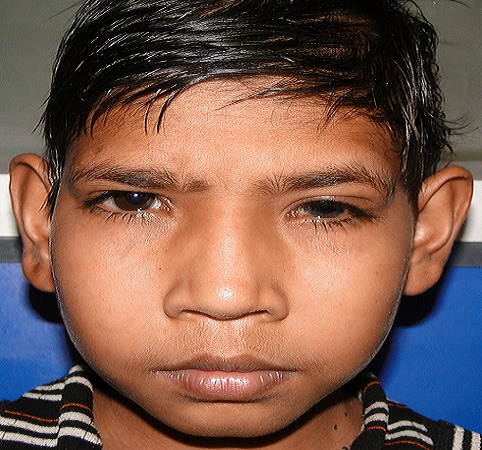
Photograph showing bilateral mild ptosis, hypertelorism and antimongoloid slant in a 13 year old boy

**Figure 2:**
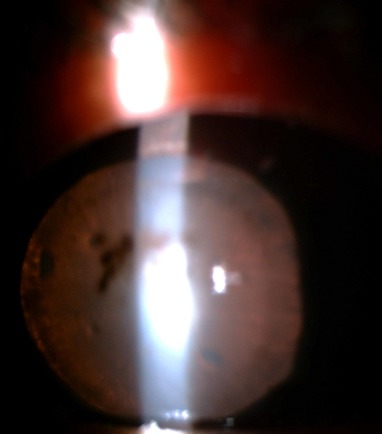
Slit lamp examination showing the crystalline lens in the anterior chamber in the left eye

A 13- year- old boy presented with photophobia, pain and redness in the left eye (LE) for 1-week. There was no history of trauma. Ocular examination showed best corrected visual acuity (BCVA) of 6/36 in the right eye (RE) and hand movement in the left eye. The child had hypertelorism with ptosis and antimongoloid slant (Figure 1). Left eye revealed circumciliary congestion and dislocation of crystalline lens into AC (Figure 2). The right eye revealed superotemporal subluxation of normal lens. The intraocular pressure was 16mm of Hg in the RE and 36mm Hg in the LE.

**Figure 3:**
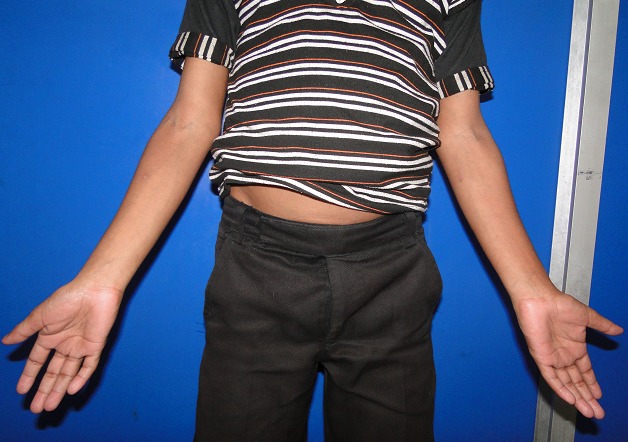
Photograph showing cubitus valgus deformity

Systemic examination revealed dysmorphic facial features with low set large ears and short webbed neck. He had short stature with cubitus valgus (Figure 3) and no secondary sex characters. There was no hepatospenomegaly and spinal changes. ECG revealed left ventricular hypertrophy with right axis deviation. Echocardiography showed patent foramen ovale with left to right shunt. The family gave a history of consanguinous marriage with normal middle aged parents and sibling. The patient gave a history of hernia repair surgery on the left side at 1 ½ years of age.

Based on systemic and ocular features, diagnosis of anteriorly dislocated lens with pupillary block glaucoma in LE and subluxated lens in RE in a case of NS was made. Karyotyping revealed normal male Karyotype. Intracapsular extraction of the dislocated lens through a corneoscleral incision was done under general anaesthesia. Postoperatively, the child was visually rehabilitated with aphakic glasses.

## Discussion

Noonan syndrome is a heterogeneous but clinically recognisable, multiple congenital anomaly syndrome. The patients were previously thought to have a form of Turner syndrome, with which NS shares numerous clinical features. The observation that patients with NS have normal karyotypes was important in allowing the distinction to be made between Turner and NS reported by Sharland M and Jamieson CR et al [5, 6]. In either case males and females are equally affected. The cardinal features of NS include unusual facies consisting of hypertelorism, antimongoloid slant, ptosis (48%) strabismus (48%), amblyopia (33%), refractive errors, epicanthal folds (61%), low set ears with thickened helices and short webbed neck. Other cardinal features include congential heart disease (in 50%), short stature and chest deformity. Anterior segment changes include prominent corneal nerves, anterior stromal dystrophy, cataracts and panuveitis found by Lee NB and Reynolds DJ et al [1, 2]. Features of neurofibromatosis type 1(NF1) like Lisch nodules which could be the leading sign for diagnosing neurofibromatosis- NS have been mentioned by Al-Fawaz AM et al [7].

Luxation of the lens to the AC usually causes complications, such as corneal edema, acute attack of glaucoma caused by a pupillary block, or an anterior uveitis. Surgical removal of a dislocated lens should be done as soon as possible suggested by Choi DY [4].

## Conclusion

It is important to determine the aetiology of luxation because the associated disorders have different requirements for monitoring and/or therapy. All patients with NS require ongoing developmental, audiologic, and ophthalmologic follow-up.
